# Addressing the medicines access challenge through balance, evidence, collaboration and transparency: key take-away lessons of the 4th PPRI Conference

**DOI:** 10.1186/s40545-021-00300-3

**Published:** 2021-01-25

**Authors:** Sabine Vogler, Nina Zimmermann, Zaheer-Ud-Din Babar, Reinhard Busse, Jaime Espin, Aukje K. Mantel-Teeuwisse, Dimitra Panteli, Fatima Suleman, Veronika J. Wirtz

**Affiliations:** 1 WHO Collaborating Centre for Pharmaceutical Pricing and Reimbursement Policies, Pharmacoeconomics Department, Gesundheit Österreich GmbH (GÖG/Austrian National Public Health Institute), Vienna, Austria; 2 University of Huddersfield, Huddersfield, UK; 3 Department of Health Care Management, Technische Universität Berlin, Berlin, Germany; 4 Andalusian School of Public Health (EASP), Granada, Spain; 5 Division of Pharmacoepidemiology & Clinical Pharmacology, Utrecht Institute for Pharmaceutical Sciences (UIPS), Utrecht University, Utrecht, The Netherlands; 6 WHO Collaborating Centre for Pharmaceutical Policy and Evidence Based Practice, Discipline of Pharmaceutical Sciences, School of Health Sciences, University of KwaZulu-Natal (Westville Campus), Durban, KwaZulu-Natal South Africa; 7 WHO Collaborating Centre in Pharmaceutical Policy, Department of Global Health, Boston University School of Public Health, Boston, MA USA

**Keywords:** Access to medicines, Policy options, Affordability, Pricing policies, Reimbursement, Medicine prices, Debate, Collaboration

## Abstract

The 4th PPRI Conference, held in Vienna in October 2019, addressed issues related to equitable and affordable access to medicines. A multi-stakeholder audience from around the globe discussed solutions and best practice models for current challenges such as high-priced medicines, limitations of current pricing and reimbursement policies and tight budgets for health technologies. A multi-faceted approach (so-called balance, evidence, collaboration and transparency/BECT strategy) was also discussed. This includes an improved balance of different interests and policy areas, generation of relevant evidence, collaboration between countries and stakeholders, and transparency, and was considered as the most promising pathway for the future.

On 23 and 24 October 2019, the 4th PPRI Conference entitled “Medicines access challenge—The value of pricing and reimbursement policies” was held in Vienna to address issues related to equitable and affordable access to medicines. More than 200 participants from around 50 countries attended the event. The conference was organised by the Vienna World Health Organization (WHO) Collaborating Centre for Pharmaceutical Pricing and Reimbursement Policies affiliated to the Pharmacoeconomics Department of Gesundheit Österreich GmbH (GÖG/Austrian National Public Health Institute) supported by an Advisory Board of experts of international institutions and national policy-makers and a Scientific Programme Committee of academics working in this area.

PPRI stands for “pharmaceutical pricing and reimbursement information” and relates to the thematic focus of the organiser’s activities. In addition, it is the name of a network coordinated by the Austrian National Public Health Institute, which comprises public authorities responsible for pricing and reimbursement of medicines in more than 50 countries as well as European institutions and international organisations such as the European Commission, the Organisation for Economic Development and Cooperation (OECD) as well as WHO [1].

Access to affordable medicines still remains a challenge in the twenty-first century. In recent years, some very expensive medicines have entered the markets, putting the long-term financial sustainability of health systems at risk. The 4th PPRI Conference aimed at discussing solutions for the challenge of affordable and equitable access to needed medicines globally. In particular, the role of pharmaceutical pricing and reimbursement policies in this context was explored. The conference provided keynote speeches, panel discussions, and oral and poster sessions.

## Value of established and innovative pricing and reimbursement policies

While keynote speakers discussed the challenge of access to medicines from different perspectives, they all attributed pharmaceutical pricing and reimbursement policies a key role as contributor to equitable and affordable access to medicines. According to the speakers, these policies will remain important in the future, but they may need to further evolve into more advanced strategies. Importantly, optimising policy options should be based on a holistic view that considers the whole life-cycle of medicines. For instance, measures related to price-setting and determining reimbursement should be considered in conjunction with policy interventions at other stages of the “value chain” such as horizon scanning or use of standard treatment guidelines. This comprehensive approach would also imply interaction and dialogue between stakeholders at different stages and within different policy areas.

In her opening keynote “Global Access to Medicines Challenge. Time for a new approach?”, Ellen t’Hoen of “Medicines Law & Policy” critically reviewed incentives for pharmaceutical industry, suggesting a “sufficiency principle” for them (“When is enough enough?”). Calling for more transparency in prices and costs, she urged for new funding models for research and development (R&D) based on delinkage models which means that financing of the development of new medicines is no longer dependent on the ability to charge high prices.

Sarah Garner, of the WHO Regional Office for Europe, explained the rationale of Universal Health Coverage (UHC): no one should be left behind. WHO supports countries to achieve the Strategic Development Goal (SDG) 3.8 on UHC (“*achieve universal health coverage (UHC), including financial risk protection, access to quality essential health care services, and access to safe, effective, quality, and affordable essential medicines and vaccines for all*” [2]) through policy dialogue, strategic support, technical assistance and service delivery. While requests from countries to WHO may differ with regard to their income level and progress in UHC, Sarah Garner reminded participants that ensuring access to high-cost medicines is also challenging for high-income countries with advanced UHC.

In the closure keynote, Suerie Moon, of the Graduate Institute of International and Development Studies in Geneva, called for more “out-of-the-box thinking”, which would include considerations of global fairness and affordability of medicines and different ways to remunerate innovation. She challenged the concern that price regulation by public authorities would imply less innovation since high prices do not necessarily maximise revenues and regulation can send important signals to the market regarding the most publicly beneficial directions of innovation. She considered an “out-of-the-box thinking” complementary to established policy options, for which a comprehensive “tool box” is already available. Suerie Moon stressed that any calibrated intervention requires an understanding of the system. Thus, information on, e.g. net medicines prices, net R&D costs, investments of sponsors, patent status and data on safety, efficacy and health system effects is needed—a clear call for more transparency in the system.

In a panel debate, some of the participating stakeholders (representing international community, national public authorities for pricing and reimbursement, patients and pharmaceutical industry) voiced similar ideas. Gaelle Krikorian of Médecins Sans Frontières (MSF) demanded that, before coming up with new solutions, an analysis of the pharmaceutical system, which is not a normal commodity market, needs to be conducted. For doing this, transparency is needed. Florian Turk of Sandoz Biopharmaceuticals (Germany) called for improvements in pharmaceutical policy-making: decision-makers should be clear about the objectives they aim to achieve and about the intended effects of the policies, which—if applied simultaneously—may be contradictory. Marcel van Raaij of the Dutch Ministry of Health, Welfare and Sports and involved in the Beneluxa Initiative [3], one of the cross-country collaborations in pharmaceutical policies [4], also highlighted the importance of good policy-making. In his view, current high medicines prices are not a market failure but a policy failure. As price (and reimbursement) negotiations are increasingly being conducted, Andrew Rintoul of WHO Headquarters encouraged governments to exert the power they have as sole buyer. “You don’t get what you deserve, but you get what you negotiate”, he commented.

**Fig. 1:**
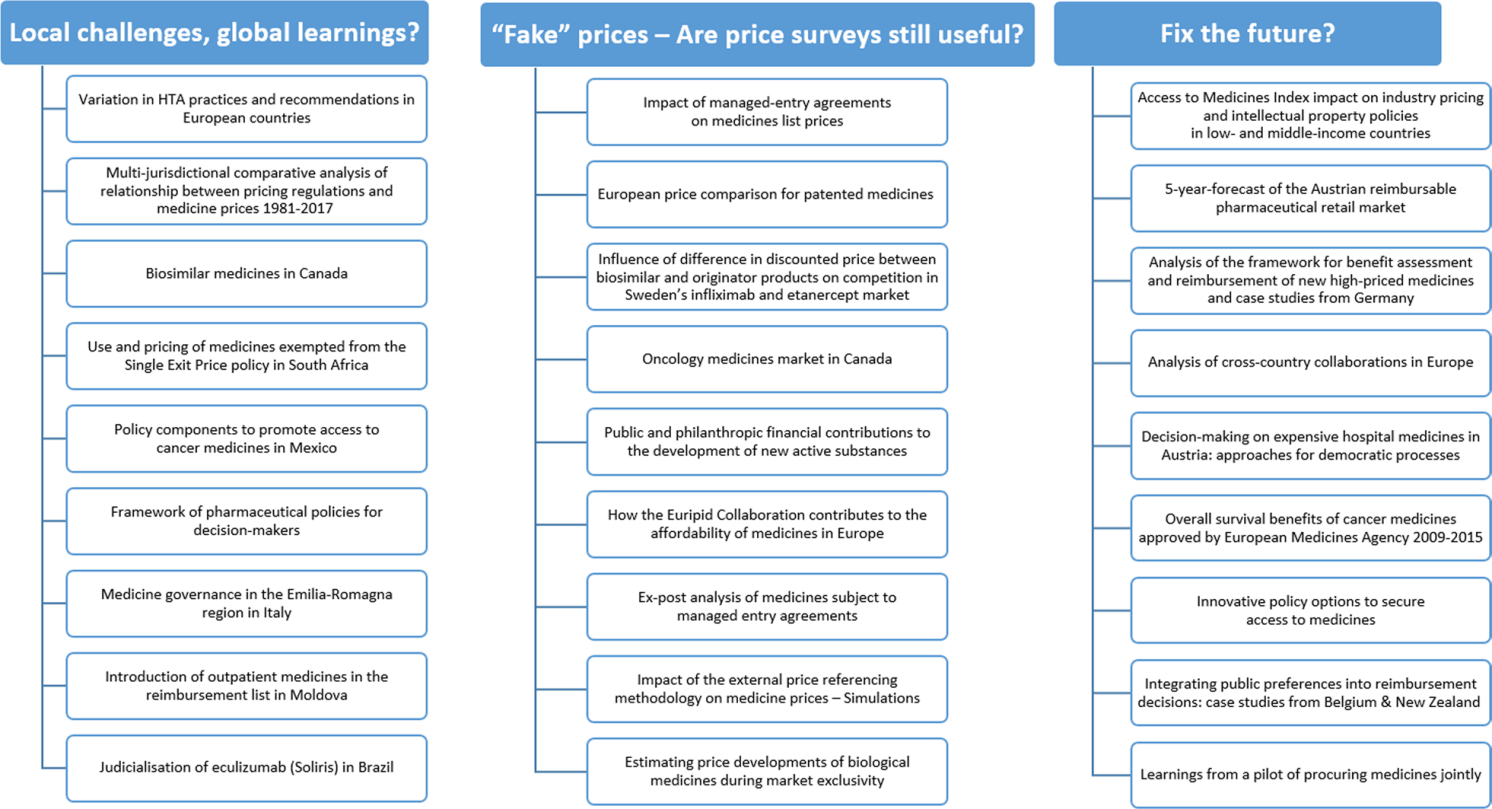
Selection of presentations and posters based on submitted and accepted abstracts. Accepted abstracts were published in the Conference Proceedings [5]

The overarching conference theme was addressed in three thematic strands, for which selected abstract submitters had been invited to present their findings in oral presentations or on posters (cf. Fig. 1 for an overview of presented abstracts [5] in the parallel sessions and Box 1 on the awards).

Box 1: PPRI Conference Awards The Scientific Programme recognised the work of researchers by granting three awards of the 4th PPRI Conference:

Young Researcher Award: For the best abstract submission by a researcher below the age of 35: *Sarah Wolf* of the Ludwig Boltzmann Institute for Health Technology Assessment (Austria) received this award for her abstract “Supporting Decision-Making on Costly Hospital Drugs in Austria: Approaches for Democratic Processes”;

Best Poster Award: *Helle Pasgaard Rommelhof, Lars Ole Madsen, Dorthe Bartels, Lise Grove and Trine Ann Behnk* of the Danish Procurement Agency Amgros I/S were recognised for their poster entitled “Joint Procurements—Learnings from a pilot of procuring medicine jointly”;

Best Presentation Award: *Rick A. Vreman* of Utrecht University (the Netherlands) was awarded for his presentation “Differences in health technology assessment recommendations between European jurisdictions: the role of practice variations”.

## More evidence to prevent the “penguin effect”

In their introductory key notes to sessions under strand 1 (Local challenges, global learnings), Veronika Wirtz (Boston University) and Jaime Espin (Andalusian School of Public Health) stressed the importance of global learning, including peer-learning. The latter allows moving beyond simple information dissemination from high-income countries to low- and middle-income countries, as lower-resourced countries also have important experiences to share. There is the risk that a policy is simply “copied” from one country to another, without having collected sufficient reliable information about its success and failures when adopted elsewhere (described as “penguin effect” in literature [6]). Managed-entry agreements were mentioned as an example of a commonly used policy option with limited evidence. As impact assessments of local policy measures are frequently missing, further countries may repeat the same mistakes.

## Price transparency to address “fake prices”

The sessions of strand 2 addressed the question as to whether, or not, price surveys and comparisons are still useful, as list prices of new medicines do not reflect discounts and rebates granted by pharmaceutical industry, as the latter usually remain confidential. While acknowledging these limitations, both key note speakers in strand 2 sessions (Dimitra Panteli of the Technical University Berlin and Inneke Van de Vijver of the Belgian Health Insurance Fund) considered the usefulness of price comparisons as a given for research and policy-making, e.g. a benchmark price to provide a first indication, or support to understand expenditure data. Therefore, price transparency should remain on the agenda, and, according to Inneke Van de Vijver, the discussion to foster transparency should be expanded to include further elements of budgeting and financing (e.g. willingness-to-pay thresholds of payers). Research findings and proposals presented in strand 2 were subject to controversial discussions, which reflected the different perspectives of the stakeholders who attended the PPRI conference. It also confirmed how topical and sensitive the issue of medicines prices is.

## Fixing the future

Input and discussions in strand 3 offered excellent complementary “food for thought” on directions for the future. Keynote speaker Valérie Paris of the Organisation for Economic Development and Cooperation (OECD) recommended to further develop policy options, including restoring trust and dialogue between industry and other stakeholders, reducing R&D cost, optimising the use of performance-based managed-entry agreements and developing and adjusting pull and push incentives to encourage innovations in areas with unmet medical needs. She also called for facilitating cooperation in HTA—a topic that was followed up by the key note delivered by Wim Goettsch of Utrecht University. He reported about ongoing work to adapt the HTA methodology to more complex and personalised medicines as part of the EU funded Horizon 2020 HTx project [7].

## The BECT approach: balance, evidence, collaboration and transparency

**Fig. 2:**
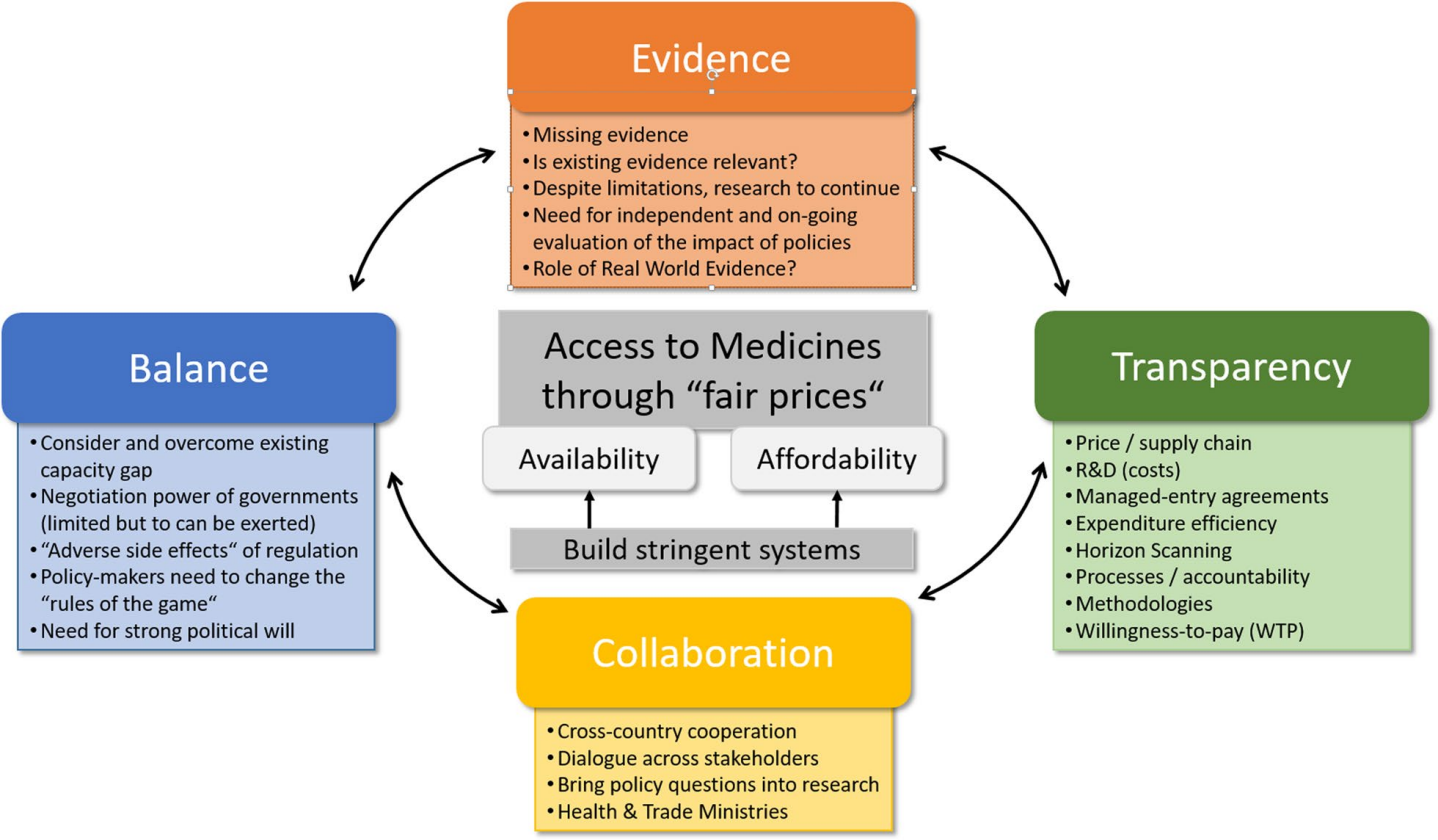
Summary of challenges for patient access to medicines and possible solutions discussed at the 4th PPRI Conference

While it was acknowledged that there is no silver bullet to ensure equitable and affordable medicines access to patients while keeping the markets attractive, competitive and sustainable, the 4th PPRI Conference identified directions for the future. The conference concluded that the “best” approach for the future would acknowledge four, somewhat interlinked, components (Fig. 2).

First, it was recommended to balance the different interests of stakeholders involved and targeted by pharmaceutical policy. This balancing endeavour should also consider and address current imbalances of power, capacity and resources between the public administration and the private sector (e.g. through capacity-building activities). To strengthen the balance in the pharmaceutical sector, more government investment and leadership is needed to identify priorities and areas for more regulation. These efforts require strong political will and good market intelligence.

Second, more relevant evidence is needed. Lack of evidence was identified with regard to, e.g. clinical assessments (including real-world situations), economic data such as “real” prices and costs (that are needed to make sound decisions). Rigorous, well-designed evaluations of the impact of policies are missing.

Third, the value of collaboration was highly acknowledged, and it was urged to foster dialogue, coordination and cooperation. This concerns different layers, including cooperation and coordination between stakeholders, between public authorities along the “value chain” (such as regulatory authorities, HTA bodies and payers), between clinical science and policy-related research and across countries.

Fourth, transparency was seen by several (but not all) conference participants as an important tool to improve access to medicines. However, it was stressed that transparency should not only be promoted with regard to medicines prices and R&D costs, but should also be seen in a broader context. This would comprise the willingness-to-pay threshold as well as methods and processes for pricing and reimbursement decisions, for instance.

A few months after the 4th PPRI Conference, which took place at the end of October 2019, the world was hit by the COVID-19 pandemic. Crisis management has been having highest priority for governments. More than ever equitable and affordable access to (new and established) medicines is needed, and the COVID-19 crisis underlines, once again, the need for balancing interests, evidence-generation, fostering collaboration and promoting transparency.

## Data Availability

Accepted abstracts were published in the PPRI Conference Supplement [5]: https://joppp.biomedcentral.com/track/pdf/10.1186/s40545-019-0194-x. The materials of the conference (presentations, abstracts [8] including country abstracts [9]) are publicly accessible for download at the PPRI Conference website: https://ppri.goeg.at/ppriconference2019. The affiliations of the mentioned speakers and panellists relate to their positions at the time of the conference.
